# Translational Research of Zoonotic Parasites: Toward Improved Tools for Diagnosis, Treatment and Control

**DOI:** 10.3390/pathogens10111416

**Published:** 2021-11-01

**Authors:** Vito Colella, Rebecca J. Traub, Robin B. Gasser

**Affiliations:** Faculty of Veterinary and Agricultural Sciences, The University of Melbourne, Parkville, VIC 3010, Australia; rebecca.traub@unimelb.edu.au (R.J.T.); robinbg@unimelb.edu.au (R.B.G.)

A range of factors, including social, demographic and economic transformation and human-induced environmental changes, are influencing the emergence or re-emergence of zoonoses, posing new challenges in how we detect, treat and prevent such diseases. Fortunately, advanced “next-generation” sequencing (NGS), ‘omic’ and immunological techniques provide improved tools to explore zoonotic pathogens; for example, the advent of portable genome sequencing technology for the detection and sequencing of the Ebola virus during recent outbreaks in West Africa facilitated and accelerated the implementation of Ebola surveillance to monitor and prevent further outbreaks. The importance of such developments is further exemplified by the rapid and widespread deployment of genomic and informatic tools in many countries for diagnosis and monitoring during the SARS-CoV-2 pandemic. Translational research of zoonotic pathogens is best achieved using a multidisciplinary approach through collaborations, with a clear focus on alleviating the impact of these pathogens and of the diseases that they cause, particularly in underprivileged communities.

This Special Issue brings together a collection of articles on aspects of zoonotic protistan and metazoan parasite translational research, providing insights into areas of epidemiology, pathogen and vector discovery, and therapy, with significant implications for the prevention and control of zoonotic diseases.

Vector-borne pathogens (VBPs) are responsible for life-threatening conditions in humans and animals. NGS-based methods have been used to genetically characterise a diverse range of VBPs, being more sensitive than conventional techniques. Recently, Huggins et al. [1] established an NGS-based approach to identify a wide range of VBP species, enhancing performance and reducing non-specific amplification from host DNA by employing ‘blocking primers’. Additionally, using NGS, Chandra et al. [2] showed that geographical origin is a key factor in shaping the microbiota of ticks, influencing the bacteria transferred to their vertebrate hosts and, potentially, disease.

Mosquitoes are major vectors that transmit a diverse array of pathogens, including arboviruses and *Plasmodium*. Dahmana et al. [3] showed how understanding the biology, distribution and vector competence of mosquitoes can assist disease surveillance and guide the development of novel strategies for the control of mosquito-borne diseases, particularly those involving insecticide-resistant vectors. Thannesberger et al. [4] employed an NGS-based approach for virome characterisation in populations of medically important mosquitoes (*Aedes aegypti* and members of the *Culex pipiens* complex) and discovered a rich viral community in a diverse range of host species. Their study recorded novel virus sequences assigned, mainly, to circular Rep-encoding single-stranded (CRESS) DNA viruses. These viruses have been demonstrated to use mosquitoes as “virus mixing vessels”, where the genetic recombination or re-assortment of diverse viruses can ensue, potentially resulting in novel pathogens that are able to jump species barriers [4]. Zittra et al. [5,6] utilised molecular methods to identify members of particular mosquito species with phenotypic differences in behaviour, physiology, hosts and habitat preferences; for instance, metabarcoding was applied to genetically characterise *Culicoides* and subterranean mosquito communities that might serve as reservoirs for significant human pathogens. On the other hand, Kniha et al. [7] combined the use of morphological methods, DNA sequencing and mass spectrometry to identify sandfly species, enabling the characterisation of *Phlebotomus mascittii* (a putative vector of *Leishmania infantum*) in metropolitan environments.

Leishmaniases and trypanosomiases are groups of vector-borne diseases caused by protistan parasites affecting millions of people and animals around the world. Hong et al. [8] prioritised the development of an accurate and cost-effective diagnostic test, as well as the use of animals as sentinels, as effective tools for the epidemiological surveillance of leishmaniases. Gulas-Wroblewski et al. [9] developed cost-effective DNA storage and DNA extraction methods for the molecular detection of *Trypanosoma cruzi* in field-based investigations. Furthermore, Zheoat et al. [10] discovered that 2′,4′-dimethoxy-6′-hydroxychalcone from a plant extract of *Polygonum salicifolium* had anti-leishmanial and anti-trypanosomal effects in the absence of toxicity against a human cell line, suggesting its potential use against *Leishmania mexicana*, *Trypanosoma brucei* and *T. congolense*.

Diagnostics is fundamental when monitoring neglected tropical diseases (NTDs), such as onchocerciasis, before, during and after mass drug administration. Hotterbeekx et al. [11] evaluated the diagnostic specificity and sensitivity of detecting microfilariae in skin snips, or specific IgG4 antibodies (anti-OV16) in ELISA or in a rapid diagnostic test (RDT) in the blood of persons with epilepsy living in an onchocerciasis-endemic region in the Democratic Republic of Congo. Of the three methods, ELISA and RDT had the highest respective sensitivities and specificities, resulting in an improved diagnosis of onchocerciasis. Similarly, Panarese et al. [12] reported an increased proportion of dogs diagnosed with *Dirofilaria immitis* (heartworm) when immune complex dissociation was applied prior to ELISA, although the specificity of this approach requires a critical assessment, as indicated by the authors [12].

Evaluating and selecting suitable tests are also critical for the diagnosis of pathogens, such as species of *Toxoplasma* and *Echinococcus*, occurring in different tissues of numerous host species and the environment. A systematic review by Liyanage et al. [13] revealed that the diagnostic performance of ELISA tests for *T. gondii* varied considerably, depending on the type of sample (serum, ‘meat juice’ and milk), antigen (native, recombinant or chimeric) and antibody-binding reagents used, with the combined use of recombinant and chimeric antigens resulting in better performance compared with native or single recombinant antigens. Bucher et al. [14] proposed two DNA extraction methods for the cost-effective detection of *Echinococcus multilocularis* using a loop-mediated isothermal amplification test, able to be applied in large-scale field studies in low-income countries. Interestingly, Rousseau et al. [15] developed a diagnostic method for the detection of DNA from the ectoparasitic mite *Sarcoptes scabiei* in the faeces of *Canis lupus signatus* (Iberian wolf). This non-invasive method was developed for the surveillance of scabies, a disease that can have a significant adverse impact on wildlife.

Soil-transmitted helminths (STHs) are a group of parasitic worms causing a major disease burden in people living in disadvantaged communities. Of these worms, *Strongyloides stercoralis* and some species of hookworms infect canids, representing reservoirs for transmission to humans. Beknazarova et al. [16] estimated the prevalence of these STHs in dogs in remote Australian communities, with the aim of guiding future intervention and prevention strategies to control these parasites in both humans and canines.

The wealth of literature published over recent decades requires critical systematic appraisals of information on NTDs. Taghipour et al. [17] reviewed available medical literature to test a previous hypothesis of a possible association between *Toxocara* infection/exposure and multiple sclerosis (MS). Despite finding an association between anti-*Toxocara* IgG serum antibodies and MS in the published literature, the authors concluded that well-designed and -controlled studies would be essential for a rigorous assessment of this association [17]. On a distinct topic, focused on the evaluation of the function of a particular molecule, Mughal et al. [18] characterised the right open reading frame protein kinase (*Sj*-*riok-1*) gene and its gene product (*Sj*-RIOK-1) in *Schistosoma japonicum* (a human blood fluke). The chemical knockdown of *Sj*-RIOK-1 caused a reduction in worm viability and the accumulation of mature oocytes in the seminal receptacle, and of spermatozoa in the sperm vesicle, suggesting an involvement in reproductive and/or developmental processes in *S. japonicum* [18].

In conclusion, we believe that this Special Issue illustrates how multidisciplinary research can deliver improved tools for exploring zoonotic parasites and encourages translational research—from bench to bedside or the field—with a focus on alleviating the impact of zoonotic parasitic diseases, particularly in disadvantaged communities around the world.
